# Prediction of Postnatal Growth Failure in Very Low Birth Weight Infants Using a Machine Learning Model

**DOI:** 10.3390/diagnostics13243627

**Published:** 2023-12-08

**Authors:** So Jin Yoon, Donghyun Kim, Sook Hyun Park, Jung Ho Han, Joohee Lim, Jeong Eun Shin, Ho Seon Eun, Soon Min Lee, Min Soo Park

**Affiliations:** 1 Department of Pediatrics, Yonsei University College of Medicine, Seoul 03722, Republic of Korea; sojinyoon@yuhs.ac (S.J.Y.);; 2 Department of Advanced General Dentistry, Yonsei University College of Dentistry, Seoul 03722, Republic of Korea; 3 InVisionLab Inc., Seoul 05854, Republic of Korea

**Keywords:** postnatal growth failure, prediction, performance, machine learning

## Abstract

Accurate prediction of postnatal growth failure (PGF) can be beneficial for early intervention and prevention. We aimed to develop a machine learning model to predict PGF at discharge among very low birth weight (VLBW) infants using extreme gradient boosting. A total of 729 VLBW infants, born between 2013 and 2017 in four hospitals, were included. PGF was defined as a decrease in z-score between birth and discharge that was greater than 1.28. Feature selection and addition were performed to improve the accuracy of prediction at four different time points, including 0, 7, 14, and 28 days after birth. A total of 12 features with high contribution at all time points by feature importance were decided upon, and good performance was shown as an area under the receiver operating characteristic curve (AUROC) of 0.78 at 7 days. After adding weight change to the 12 features—which included sex, gestational age, birth weight, small for gestational age, maternal hypertension, respiratory distress syndrome, duration of invasive ventilation, duration of non-invasive ventilation, patent ductus arteriosus, sepsis, use of parenteral nutrition, and reach at full enteral nutrition—the AUROC at 7 days after birth was shown as 0.84. Our prediction model for PGF performed well at early detection. Its potential clinical application as a supplemental tool could be helpful for reducing PGF and improving child health.

## 1. Introduction

Although medical advances have improved the overall quality of care of preterm infants, postnatal growth failure (PGF) in preterm infants is still a challenging problem [1]. The prevalence of growth failure in preterm infants at discharge from the neonatal intensive care unit (NICU) was observed at approximately 25–50%, although the definition of growth failure may vary. Growth failure at discharge may indicate an increased risk of long-term health problems, especially developmental deficits, so early detection and intervention are important to prevent this and promote optimal growth and development. In addition to treating the underlying medical illness, health care professionals may regularly assess growth and provide interventions such as adjusting feeding schedules, but a more systematic approach may be needed.

Several risk factors have been identified to increase PGF in preterm infants using conventional logistic regression. Preterm infants with low gestational ages are a high-risk group for PGF after adjusting for co-morbidity in preterm infants [2]. Male, small for gestational age (SGA), preterm infants who were on invasive ventilator care on the first day of life, requiring respiratory support at 28 days of age, and receiving steroid therapy during hospitalization were reported to be particularly at risk of PGF [3,4]. Accompanying medical conditions were significantly associated with PGF; respiratory distress syndrome (RDS), necrotizing enterocolitis (NEC), spontaneous intestinal perforation, sepsis, and retinopathy of prematurity showed especially significant relationships [1,5]. Nutritional factors such as protein intake, enteral nutrition, breastfeeding, and parenteral nutrition play a crucial role in the postnatal growth of preterm infants; a multidisciplinary approach using a nutritional support team adjusts nutritional regimens as needed to promote optimal growth. However, previous results generated using regression analysis show the need to interpret relationships cautiously because of limited generalizability, assumption of linearity, lack of causal relationships, non-stationarity, multicollinearity, and small sample size [2,3,4,5].

Machine learning has been widely applied in the medical field due to its ability to process large amounts of data, identify patterns, and make predictions based on complex relationships between variables. In the field of neonatology, researchers have explored the prediction of disease progression and outcomes at early stages, focusing on conditions like apnea of prematurity, sepsis, and mortality. Previous studies predominantly examined limited time points, such as at birth [6,7,8]. However, there is a scarcity of research addressing the extended duration and complex circumstances of diseases in preterm infants through the application of machine learning. Additionally, efforts to enhance predictive performance involve modifying clinical data, incorporating methods such as wrapper-based feature selection, and feature addition [8,9,10].

Early prediction and prevention of PGF in preterm infants will be crucial for their long-term neurodevelopmental prognoses. The presence of variance across hospitals and clinics in the application of known risk factors is a common challenge in clinical fields. This variability can have significant implications for patient care, outcomes, and the overall quality of healthcare. Automated prediction using machine learning is deemed beneficial in light of risk variables that exist from birth, as well as newly emerging morbidity and progression over the postnatal clinical course.

Only a few studies have been conducted with the aim to predict PGF using machine learning. This research group noted that the extreme gradient boosting (XGB) algorithm showed the best performance for predicting PGF in all six metrics using the database of the Korean Neonatal Network (KNN) between 2013 and 2017, when four different machine learning models (XGB, random forest, support vector machine, convolutional neural network) and multiple logistic regression models were compared at different time points (at birth; at 7 days and after 14 days; and after 28 days of life). XGB showed a better performance compared to MLR (*p* = 0.03) with AUROC (0.74) at Day 7. The accuracy of prediction increased slightly at Day 7 compared to birth, but plateaued thereafter [11]. While previous research has shown the potential for a predictive model in assessing PGF among preterm infants using machine learning, persistent concerns about the model’s performance underscore the need for ongoing improvements. New research is essential to significantly enhance the accuracy of the PGF prediction model.

Our objective was to develop the best machine learning model, using feature selection and addition, to predict postnatal growth failure at discharge in VLBW infants during their NICU stay.

## 2. Materials and Methods

**Figure 1:**
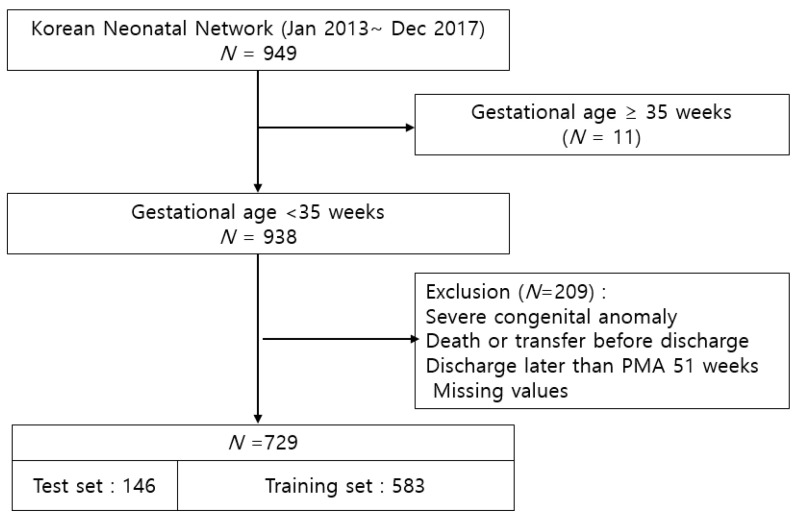
Study population.

A total of 949 VLBW infants, born between 2013 and 2017 in four hospitals in Korea (Gangnam Severance Hospital in Seoul, Severance Hospital in Seoul, Chonnam National University Hospital in Gwangju, and Gangnam Cha University Gangnam Medical Center in Seoul) participating in the KNN, were included. The KNN is a prospective web-based registry for VLBW infants using a standardized electronic case report form in South Korea, and it collects data including maternal information, delivery information, neonatal information, diagnosis, treatment, and morbidities [12]. The exclusion criteria of the current study were as follows: infants with gestational age (GA) >35 weeks; infants with severe congenital anomalies; infants who died before discharge or were discharged later than a postmenstrual age of 51 weeks; infants who were transferred to other hospital; and infants who had missing values. For the Fenton growth charts available for those under 51 weeks of postmenstrual age (PMA), infants who were discharged later than PMA 51 weeks or who had missing values were excluded. Finally, a total of 729 infants were included in this study (Figure 1).

The KNN registry was approved by the institutional review boards of all participating hospitals. Informed consent forms were signed by the parents of the infants during enrollment in the KNN. The present study was performed in accordance with the ethical standards of the 1964 Declaration of Helsinki and its later amendments, and was approved by the KNN data management committee and the Gangnam Severance Hospital IRB (IRB 3-2021-0329).

The following definitions were guided by the manual of operations of the KNN. SGA was defined as a birth weight lower than the 10th percentile for gestational age (GA) according to Fenton’s growth chart [13]. PGF was defined as a decrease in the z-score of weight between birth and discharge of more than −1.28 using Fenton’s growth chart [7]. Maternal hypertension was defined as newly diagnosed hypertension in a pregnant woman after 20 weeks of gestation. Prolonged rupture of membranes (PROM) was defined as an 18 h or longer duration of the rupture of membrane. Air leak syndrome was defined as a disease entity including pneumothorax, pneumomediastinum, and pulmonary interstitial emphysema that needed invasive procedures, such as the insertion of a chest tube or needle aspiration. Respiratory distress syndrome (RDS) was defined as respiratory failure due to primary surfactant deficiency. Medically treated patent ductus arteriosus (PDA) was treated through medication, and surgical treatment of PDA was treated with surgical ligation. Severe intraventricular hemorrhage (IVH) was defined as grade 3 or 4 IVH based on cranial imaging performed before 28 days of life [14]. NEC was defined according to the modified Bell’s criteria [15]. Sepsis was defined using blood cultures positive for bacteria, or fungi and antibiotic therapy for ≥5 days. Non-invasive ventilation was defined as the use of any non-invasive positive pressure support, including continuous positive airway pressure and high flow nasal cannula at each time point. Parenteral nutrition (PN) was present if parenteral nutrition was supplied at each time point, and full enteral nutrition (EN) was present if EN above 100 mL/kg was supplied at each time point. Postnatal weight was recorded daily from birth during the NICU stay by the clinical staff in charge of care. Weight was transformed into z-scores in reference to Fenton’s growth chart, for comparison with the population of the same gestational or postmenstrual age at each time point.

This study was conducted to find the best machine learning algorithm for the prediction of PGF at four time points—at birth (Day 1), 7 days after birth (Day 7), 14 days after birth (Day 14), and 28 days after birth (Day 28)—using the XGB model. In machine learning algorithms, feature selection is crucial for improving classification accuracy and minimizing the amount of features [16]. In order to improve the performance of the selected model, we used the feature selection method to select variables that have high contributions to prediction among the initially selected variables. Lastly, we developed a model with further improvement in performance by adding clinical information to the model.

The dataset was divided into 5 datasets through stratified fivefold cross validation and trained at a ratio of 4:1, and the average value of the result accuracy was presented. Fivefold cross validation was performed because the dataset was not large. To compare the baseline demographics between the training and test sets, a chi-squared test for categorical variables and an independent two-sample *t*-test for continuous variables were used. *p*-values < 0.05 were considered statistically significant. The analysis was conducted using SPSS version 23.0 (IBM Corporation, Armonk, NY, USA). For comparison of predictive performances between the models, we used the bootstrap method to calculate *p*-values, which means that 1000 datasets, allowing for duplication, were randomly extracted and analyzed. The machine learning model’s performance was assessed using Python, employing metrics such as the area under the receiver operating characteristic curve (AUROC), accuracy, precision, sensitivity, specificity, and F1 score. The evaluation was conducted within the Anaconda distribution (Python version 3.7, https://www.anaconda.com accessed on 1 July 2021; Anaconda Inc., Austin, TX, USA) and utilized the XGBoost package, version 0.90 (https://xgboost.readthedocs.io accessed on 1 July 2021). The XGBoost algorithm, a widely used gradient-boosting framework for supervised learning tasks, was employed. Feature importance in XGBoost involves assigning scores to each feature in the input data, indicating their relative importance in predicting the target variable. Variable reduction was carried out using the Python scikit-learn library (version 1.1), with the XGB module employed to assess performance changes. No separate normalization or outlier processing was performed on XGBoost’s learning data, missing data was excluded in advance, and learning was performed with all data present. For parameter tuning, we tried to find the optimal parameters by adjusting min_child_weight, max_depth, gamma, colsample_bytree, and alpha, and found the optimal value for each parameter through local optimization (max_depth = 2, min_child_weight = 0.8, gamma = 0.2, colsample_bytree = 0.8, reg_alpha = 0.01). Also, since the amount of positive data is relatively small compared to the negative data, we attempted to increase accuracy by training with a weight of scale_pos_weight = 0.8. As for the convergence criteria for learning, 2000 steps was set as the maximum learning period, and the early stopping condition was set to stop training if the loss of the validation date did not decrease further within the previous 100 steps. We reduced the variables one by one until essential performance was maintained.

## 3. Results

**Figure 2:**
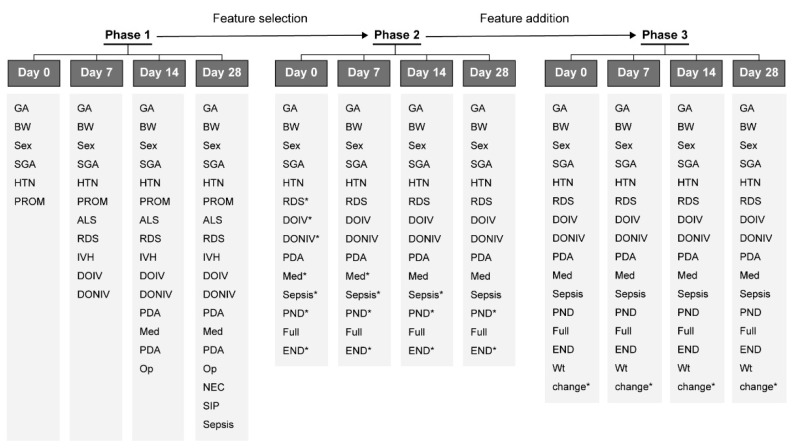
Brief explanation about the features used in each phase. Abbreviations: GA, gestational age; BW, birth weight; SGA, small for gestational age; HTN, maternal hypertension; PROM, premature rupture of membrane; ALS, air leak syndrome; RDS, respiratory distress syndrome; IVH, severe intraventricular hemorrhage; DOIV, duration of invasive ventilation; DONIV, duration of non-invasive ventilation; PDA Med, medically treated patent ductus arteriosus; PDA Op, surgical ligation of patent ductus arteriosus; NEC, necrotizing enterocolitis; SIP, spontaneous intestinal perforation; PND, duration of parenteral nutrition; Full END, full enteral nutrition day; Wt change, percentile of weight change to birth weight; * newly added feature compared to previous phase.

The baseline demographics of the training set and the test set showed no significant differences (Table 1). The predictive model of PGF at discharge was developed at four time points, including Day 1, Day 7, Day 14, and Day 28 after birth. A brief explanation about the features used in each phase is shown in Figure 2. In the phase 1 model, variables from the KNN dataset which, through MLR analysis, were shown to exhibit significant associations with PGF at the respective time points were selected. In phase 2, using the ranked feature importance, a total of 12 features were selected in order of most significant contribution and identically applied to each time point. In addition, to improve the performance, nutritional features including parenteral nutrition (PN) and full enteral nutrition (EN), which can affect growth, were added. In phase 3, after adding the weight change feature from birth weight to each time point, the predictive performance was analyzed. A total of 13 features, including sex; gestational age; birth weight; SGA; HTN; RDS; duration of invasive ventilation; duration of non-invasive ventilation; medically treated PDA; sepsis; PN duration; full EN days; and weight change were included.

**Figure 3:**
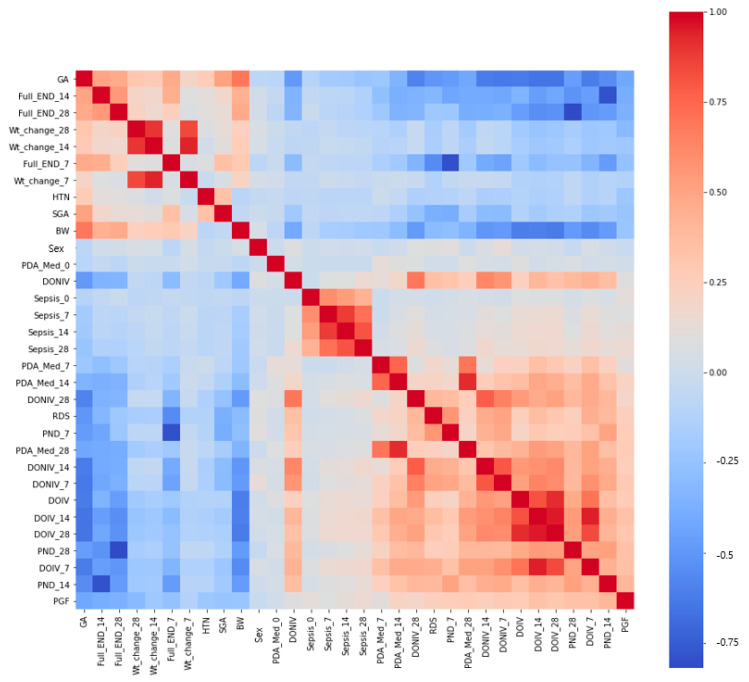
Schematic diagram of the relationships between the factors in the final model. Heatmap of the features’ correlation. Red represents a positive correlation, and blue represents a negative correlation. A darker color indicates a higher correlation, while a lighter color indicates a lower correlation. Abbreviations: GA, gestational age; SGA, small for gestational age; BW, birth weight; HTN, maternal hypertension; DOIV, duration of invasive ventilation; RDS, respiratory distress syndrome; DONIV, duration of non-invasive ventilation; PDA Med, medically treated patent ductus arteriosus; Full END, full enteral nutrition day; PND, duration of parenteral nutrition.

**Figure 4:**
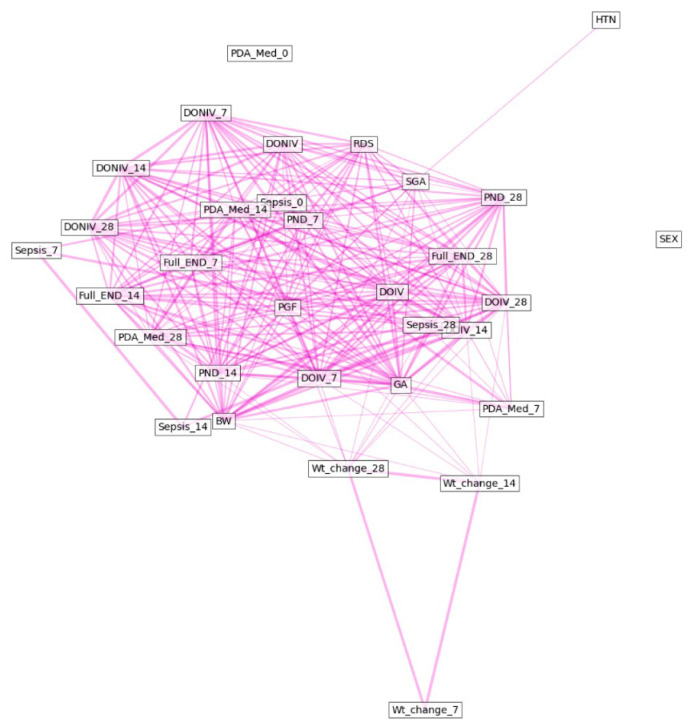
Schematic diagram of the relationships between the factors in the final model. Factors with low correlations are separated from each other and factors with high correlations are brought closer together. Abbreviations: GA, gestational age; SGA, small for gestational age; BW, birth weight; HTN, maternal hypertension; DOIV, duration of invasive ventilation; RDS, respiratory distress syndrome; DONIV, duration of non-invasive ventilation; PDA Med, medically treated patent ductus arteriosus; Full END, full enteral nutrition day; PND, duration of parenteral nutrition.

Figure 3 and Figure 4 show the relationships between each variable in the final model. The heatmap shows the feature correlation, with red indicating positive correlations and blue indicating negative correlations. A darker color indicates a higher correlation, while a lighter color indicates a lower correlation. The diagram of the relationships among the factors shows that factors with low correlations are separated from each other and factors with high correlations are brought closer together.

Table 2 shows the predictive performance after feature selection and addition using the XGB model. The performance of the model was improved after feature selection and addition compared to the phase 1 model. The 7-day AUROC was improved from 0.78 to 0.84, and the sensitivity from phase 2 to phase 3 was improved from 0.69 to 0.71. For Day 7, the final phase showed better performance in terms of the AUROC (0.84), sensitivity (0.71), and accuracy (0.76) compared to the AUROC (0.76), sensitivity (0.59), and accuracy (0.73) of phase 1. Also, this model showed a better performance at every time point.

## 4. Discussion

Using machine learning, we were able to build a novel tool to predict PGF at birth, 7 days after birth, 14 days after birth, and 28 days after birth, with a high degree of sensitivity and specificity. To our knowledge, this is the first model built with the purpose of detecting PGF to assist in clinical decision making. PGF in preterm infants is strongly associated with developmental deficits leading to long-term cognitive, behavioral, and physical problems [17]. This predictive model can be helpful for a nutritional support team in the early detection of infants with PGF.

A previous study compared the XGB model with a traditional MLR model and three machine learning models (random forest, support vector machine, convolutional neural network model) and found that the XGB model showed the best performance in predicting PGF at discharge using 11 variables at 7 days after birth [11]. However, due to a lack of informative data and variance of variables across time points, the predictive performance showed an AUROC of just below 0.75. This study was conducted to improve the predictive performance. In order to optimize machine learning models, it is important to consider various factors, including hyperparameter tuning, model complexity, and the risk of overfitting. More complex models can improve performance, but can also lead to overfitting. It has been reported that investigating the significance of variables can improve the accuracy of predicting the progression of sepsis-induced coagulopathy [18]. Another study about predicting the likelihood of acute kidney injury found that proper variable selection and feature engineering was crucial for improving the performance of the machine learning models, as including irrelevant or redundant features could negatively impact their accuracy [19]. Therefore, in this study, we carefully selected and preprocessed the input data, controlled overfitting, and simplified the data to apply equally to all time points. As for the feature selection, experts provided insight into which features are likely to be important, and analyzed the correlation between features. Furthermore, we have adopted a method of feature importance in an effort to prioritize the removal of variables that have the least impact on the learning model. To mitigate the risk of overfitting, fivefold validation, which involves splitting the dataset in various ways for training, was employed. Additionally, we have utilized techniques such as early stopping and alpha regulation to reduce the bias within the learning model itself.

The predictive power for PGF at discharge was expected to increase when the preterm infants got closer to discharge; however, no consistent findings were observed. In previous studies, the variables predicting PGF were set to gradually increase from 6 to 16 on days 0, 7, 14, and 28 after birth, but the performance index AUROC and sensitivity were the highest on the 7th day after birth, when 11 variables were selected [11]. Other studies have shown that when using a wrapper-based feature selection method, the accuracy of machine learning models for predicting the risk of developing pediatric asthma, as well as predicting clinical outcomes in pediatric patients with a traumatic brain injury, improved by 5.8% and 2.9%, respectively, compared to using all features [20,21]. In this study, the data of phase 1 showed that there was a chance that having too many features could lead to overfitting. Also, when feature importance was examined to select the valuable features at phase 2, the results for Day 7 showed an AUROC of 0.84, which was lower than the 0.85 of Day 14; but the sensitivity was 0.71, which was higher than the 0.69 of Day 14.

Adding certain informative features can improve the performance of the model. A previous study, which investigated the use of a machine learning model for predicting the risk of ADHD or pediatric sepsis, has shown the results of adding demographic features or laboratory features compared to using only clinical features [22,23]. In a study about a machine learning model used to predict the risk of pediatric asthma exacerbation, adding environmental features—such as air quality and temperature—improved the performance of the model compared to using only clinical features [24]. This study added the weight change percentile compared to birth weight, which was considered a specific informative feature, as a prediction of postnatal growth. Consequently, its utility as a support tool for predicting PGF is higher.

The incidence of PGF has increased with lower gestational age and birth, although there are variations according to the definition of PGF [3]. Moreover, PGF in SGA infants was considered the continuation of a process previously affecting fetal growth in the uterus [25]. The World Health Organization (WHO) recommends using z-scores to show anthropometric data; it appears that since 2005, reported changes in z-scores have gained increased traction [26]. Therefore, in this study, we defined PGF as a condition where the difference between the birth weight z-score and the weight z-score at discharge was above 1.28 using Fenton’s growth chart [2,13,27]. PGF babies had a higher percentage of initial weight loss and a longer duration to regain birth weight [2]. Due to the physiological weight loss in the early postnatal period of preterm infants and the delay to regain their birth weight due to neonatal morbidities, it takes these infants the first 2 weeks of life to regain their birth weight. Infants with a lower birth weight z-score experienced a greater change in their weight z-score during the first 3 days of hospitalization [25]. This finding suggests that early nutritional interventions may be particularly important for infants with lower birth weight z-scores. Therefore, it is important to focus on predicting PGF at 7 days of life.

Insufficient nutritional support was correlated with PGF. An aggressive nutritional support concept was introduced to the NICU, and encouraged support for protein accretion and growth. Recent guidelines have recommended early feeding and protein supply for the first day after birth, and a fast advance to full enteral feeding [1]. PGF preterm infants showed significant differences in the age of first and full enteral feeding, duration of PN, and lipid emulsions [28]. The PN duration and the day of reaching maximal enteral feeding were included as important features in this study. A decrease in the maximum amino acid and the average lipid during the first postnatal week was associated with the probability of poor weight gain [29]. However, due to the nature of the database from KNN, only full EN days and PN duration data were used in this study. 

This study had some limitations. First, the protocols for overall treatment and nutritional support of preterm infants could not be standardized and controlled for the analysis, due to the database being built by gathering data from multiple NICUs. Second, another significant drawback of the KNN database was the absence of more comprehensive nutritional information, including information on the types and timings of enteral feeding, the use of fortifiers, total durations, and the compositions of parenteral nutrition. Third, the study was limited to PGF at discharge, since the definition of PGF may vary. Fourth, external validation was not performed. Therefore, for a more accurate performance evaluation, a future study for external validation needs to be carried out with data not used when training the model. However, this study still offers immense significance, as we demonstrated the possibility of a predictive model for PGF of preterm infants using a machine learning technique.

The main strength of this study is that it improved the performance of the PGF prediction model by applying daily weight measurements of newborns admitted to the NICU, taking a step forward from developing a predictive model for PGF in VLBW infants. It is expected to be clinically useful if an algorithm supplemented with nutritional information is applied in future studies, to improve the limitations of the current study. By carefully selecting and pre-processing the input data, machine learning models can provide accurate and reliable predictions that can help clinicians make informed decisions regarding patient care.

## 5. Conclusions

With the improvement in the survival of preterm infants, there has been a growing focus on postnatal growth and its association with neurodevelopmental prognoses. This study developed a predictive model for postnatal growth failure at discharge in VLBW infants during their NICU stay. The model employed the XGB algorithm with feature selection and addition techniques, demonstrating robust performance in detecting PGF at discharge across various time points, including birth, 7 days, 14 days, and 28 days after birth. The potential application of this predictive model in real-world scenarios holds promise, offering support for clinical decision making regarding the early detection and implementation of aggressive interventions for PGF. This, in turn, is anticipated to contribute significantly to the improvement of neurodevelopmental outcomes in VLBW infants. Future work can be carried out to validate this model and confirm predictive performance. Also, another approach could be to develop the model’s complexity and optimize the model.

## Figures and Tables

**Table 1:** Baseline demographics of the training set and the test set of the final models.

	Training *N* = 583	Test *N* = 146	*p*-Value
Male, *N* (%)	298 (51.1)	73 (50.0)	0.810
Maternal hypertension, *N* (%)	144 (24.7)	38 (26.0)	0.740
Gestational age, mean (SD)	29.2 (2.6)	29.6 (2.5)	0.162
Birth weight (g), mean (SD)	1141.2 (250.1)	1152.6(246.8)	0.620
SGA, *N* (%)	116 (19.9)	30 (20.5)	0.861
Respiratory distress syndrome, *N* (%)	487 (83.5)	114 (78.1)	0.122
Duration of invasive ventilation until 7 days of age, mean (SD)	10.3 (17.3)	8.1 (15.1)	0.120
Duration of non-invasive ventilation until 7 days of age, mean (SD)	14.6 (15.4)	13.9 (16.2)	0.533
Medically treated PDA, *N* (%)	160 (27.4)	36 (24.7)	0.497
Sepsis, *N* (%)	53 (9.1)	13 (8.9)	0.944
PN duration, mean (SD)	24.9(20.4)	23.9(20.4)	0.623
Full EN days, mean (SD)	23.5(19.1)	22.4(19.7)	0.559
PGF, *N* (%)	205 (35.2)	49 (33.6)	0.716

**Table 2:** Comparison of the predictive performance of the XGB model among three different models.

	Day 1			Day 7			Day 14			Day 28		
	Phase 1	Phase 2	Final	Phase 1	Phase 2	Final	Phase 1	Phase 2	Final	Phase 1	Phase 2	Final
AUROC	0.76	0.79	0.79	0.76	0.78	0.84	0.77	0.80	0.85	0.75	0.77	0.93
Accuracy	0.69	0.73	0.73	0.73	0.74	0.76	0.71	0.73	0.75	0.68	0.70	0.86
Precision	0.53	0.59	0.59	0.59	0.60	0.63	0.58	0.59	0.61	0.52	0.54	0.77
Sensitivity	0.57	0.67	0.67	0.59	0.69	0.71	0.53	0.69	0.69	0.59	0.74	0.82
Specificity	0.74	0.76	0.76	0.79	0.76	0.78	0.80	0.75	0.77	0.72	0.68	0.88
F1-score	0.55	0.63	0.63	0.59	0.64	0.67	0.55	0.64	0.65	0.55	0.62	0.79

AUROC: area under the receiver operating characteristic curve.

## Data Availability

The datasets generated and/or analyzed during the current study are available from the corresponding author upon reasonable request.
